# Supplementary material for: Amplitude modulations of cortical sensory responses in pulsatile evidence accumulation
Source: eLife. 2020 Dec 2;9:e60628. doi: 10.7554/eLife.60628 (PMC7811404; doi:10.7554/eLife.60628)
Supplement: Supplementary file 1. [file elife-60628-supp1.docx]

|  | **Layers 2/3** | | | | | | **Layer 5** | | | | | |
| --- | --- | --- | --- | --- | --- | --- | --- | --- | --- | --- | --- | --- |
|  | **V1** | **AM** | **PM** | **MMA** | **MMP** | **RSC** | **V1** | **AM** | **PM** | **MMA** | **MMP** | **RSC** |
| **# sessions** | 9 | 18 | 11 | 15 | 8 | 30 | 8 | 12 | 6 | 9 | 7 | 12 |
| **# mice imaged** | 4 | 8 | 6 | 9 | 4 | 8 | 4 | 7 | 3 | 8 | 4 | 6 |

[**Supplementary File 1.**](https://docs.google.com/document/d/12frnsGqVL-UhMKL1jXzYlvriQBX_6Um6FCJjONLu834/edit#sptab_sessionMice)  Number of imaging sessions and mice for various areas and layers, for the main experiment.
