# Supplementary material for: Amplitude modulations of cortical sensory responses in pulsatile evidence accumulation
Source: eLife. 2020 Dec 2;9:e60628. doi: 10.7554/eLife.60628 (PMC7811404; doi:10.7554/eLife.60628)
Supplement: Supplementary file 2. — Mice of the Thy1 GP5.3 strain have names starting with ‘gp’, and those from the Ai93-Emx1 strain have names starting with ‘ai’ (see Materials and methods). [file elife-60628-supp2.docx]

|  |  | **Layers 2/3** | | | | | | **Layer 5** | | | | | |
| --- | --- | --- | --- | --- | --- | --- | --- | --- | --- | --- | --- | --- | --- |
|  | **% correct** | **V1** | **AM** | **PM** | **MMA** | **MMP** | **RSC** | **V1** | **AM** | **PM** | **MMA** | **MMP** | **RSC** |
| **gp31** | 73.0 | 1 | 1 | 1 | 1 | 0 | 2 | 1 | 1 | 1 | 1 | 1 | 1 |
| **gp36** | 75.3 | 2 | 1 | 1 | 1 | 1 | 2 | 2 | 1 | 1 | 1 | 1 | 1 |
| **gp37** | 70.1 | 0 | 1 | 0 | 1 | 1 | 1 | 0 | 0 | 0 | 1 | 0 | 0 |
| **gp40** | 67.7 | 0 | 2 | 0 | 0 | 0 | 9 | 0 | 1 | 0 | 0 | 0 | 5 |
| **gp42** | 67.1 | 0 | 1 | 0 | 2 | 3 | 4 | 0 | 1 | 0 | 1 | 2 | 1 |
| **gp46** | 67.8 | 0 | 0 | 1 | 3 | 0 | 4 | 0 | 0 | 0 | 1 | 0 | 2 |
| **ai50** | 68.7 | 0 | 2 | 1 | 3 | 0 | 7 | 0 | 1 | 0 | 2 | 0 | 2 |
| **ai53** | 67.8 | 2 | 0 | 0 | 1 | 0 | 0 | 2 | 0 | 0 | 0 | 0 | 0 |
| **ai55** | 71.6 | 0 | 3 | 0 | 2 | 0 | 1 | 0 | 3 | 0 | 1 | 0 | 0 |
| **ai56** | 71.9 | 4 | 7 | 6 | 1 | 3 | 0 | 3 | 4 | 4 | 1 | 3 | 0 |
| **ai57** | 67.2 | 0 | 0 | 1 | 0 | 0 | 0 | 0 | 0 | 0 | 0 | 0 | 0 |

[**Supplementary File 2.**](https://docs.google.com/document/d/12frnsGqVL-UhMKL1jXzYlvriQBX_6Um6FCJjONLu834/edit#sptab_sessionPerform)  Overall performance and number of imaging sessions for the main experiment, per mouse (rows), in various areas and layers (columns). Mice of the Thy1 GP5.3 strain have names starting with “gp”, and those from the Ai93-Emx1 strain have names starting with “ai” (see Methods).
