# Supplementary material for: Amplitude modulations of cortical sensory responses in pulsatile evidence accumulation
Source: eLife. 2020 Dec 2;9:e60628. doi: 10.7554/eLife.60628 (PMC7811404; doi:10.7554/eLife.60628)
Supplement: Transparent reporting form [file elife-60628-transrepform.docx]

***eLife’s* transparent reporting form**

We encourage authors to provide detailed information *within their submission* to facilitate the interpretation and replication of experiments. Authors can upload supporting documentation to indicate the use of appropriate reporting guidelines for health-related research (see [EQUATOR Network](http://www.equator-network.org/%20)), life science research (see the [BioSharing Information Resource](https://biosharing.org/)), or the [ARRIVE guidelines](http://www.plosbiology.org/article/info:doi/10.1371/journal.pbio.1000412) for reporting work involving animal research. Where applicable, authors should refer to any relevant reporting standards documents in this form.

If you have any questions, please consult our Journal Policies and/or contact us: [editorial@elifesciences.org](mailto:editorial@elifesciences.org).

**Sample-size estimation**

- You should state whether an appropriate sample size was computed when the study was being designed
- You should state the statistical method of sample size computation and any required assumptions
- If no explicit power analysis was used, you should describe how you decided what sample (replicate) size (number) to use

Please outline where this information can be found within the submission (e.g., sections or figure legends), or explain why this information doesn’t apply to your submission:

We aimed to record neural activity from a total number of mice that is comparable to other related neurophysiology experiments. In order to maximize the yield per animal and thus reduce statistical uncertainty as well as the number of required subjects, we then proceeded to record from as many brain regions and behavioral sessions as possible per mouse, given its willingness to engage in the behavior and the optical quality of its cranial implant. The numbers of mice and brain regions imaged are listed in Supplemental Tables 1 and 2, and the numbers of neurons analyzed are listed in the Results section.

**Replicates**

- You should report how often each experiment was performed
- You should include a definition of biological versus technical replication
- The data obtained should be provided and sufficient information should be provided to indicate the number of independent biological and/or technical replicates
- If you encountered any outliers, you should describe how these were handled
- Criteria for exclusion/inclusion of data should be clearly stated
- High-throughput sequence data should be uploaded before submission, with a private link for reviewers provided (these are available from both GEO and ArrayExpress)

Please outline where this information can be found within the submission (e.g., sections or figure legends), or explain why this information doesn’t apply to your submission:

Biological replication was performed by running the same experiment (behavioral task and neural imaging setup) across multiple mice as well as multiple experimental sessions per mouse, across days. We had some degree of technical replication by training mice on the same task using different training rigs (not equipped with microscopes). However, we only had one microscope-equipped rig for acquiring the neurophysiology data in this paper. The numbers of sessions that we repeated experiments per mouse are listed in Supplementary Table 1, and were the maximum possible given the willingness of each mouse to continue engaging in the behavior, as well as the health of its cranial implant. The only imaging session data that were omitted from analysis were those where the mouse performed poorly as explained in the Methods, indicating behavioral disengagement. For the included sessions, a small fraction of trials were discarded for fitting the amplitude-modulation models (see Methods section “Amplitude modulation models”). This trial selection was based on purely behavioral criteria, and served the purpose of retaining trials that had sufficiently similar navigational behavior so that we could correlate neural activity to behavior across these trials.

**Statistical reporting**

- Statistical analysis methods should be described and justified
- Raw data should be presented in figures whenever informative to do so (typically when N per group is less than 10)
- For each experiment, you should identify the statistical tests used, exact values of N, definitions of center, methods of multiple test correction, and dispersion and precision measures (e.g., mean, median, SD, SEM, confidence intervals; and, for the major substantive results, a measure of effect size (e.g., Pearson's r, Cohen's d)
- Report exact p-values wherever possible alongside the summary statistics and 95% confidence intervals. These should be reported for all key questions and not only when the p-value is less than 0.05.

Please outline where this information can be found within the submission (e.g., sections or figure legends), or explain why this information doesn’t apply to your submission:

Common statistical criteria and definitions used across analyses are described in the Methods section “General statistics”. The values of these statistical measures are stated in the legends of all figures.

(For large datasets, or papers with a very large number of statistical tests, you may upload a single table file with tests, Ns, etc., with reference to sections in the manuscript.)

**Group allocation**

- Indicate how samples were allocated into experimental groups (in the case of clinical studies, please specify allocation to treatment method); if randomization was used, please also state if restricted randomization was applied
- Indicate if masking was used during group allocation, data collection and/or data analysis

Please outline where this information can be found within the submission (e.g., sections or figure legends), or explain why this information doesn’t apply to your submission:

The selection of mice and strains are described in the Methods section “Experimental subjects”, with data in Supplementary File 2.

**Additional data files (“source data”)**

- We encourage you to upload relevant additional data files, such as numerical data that are represented as a graph in a figure, or as a summary table
- Where provided, these should be in the most useful format, and they can be uploaded as “Source data” files linked to a main figure or table
- Include model definition files including the full list of parameters used
- Include code used for data analysis (e.g., R, MatLab)
- Avoid stating that data files are “available upon request”

Please indicate the figures or tables for which source data files have been provided:

Except for the purely conceptual Figure 5, all figures have source data used to make distribution plots, as well as computed statistics (superset of those cited in the legend). Supplementary tables are in editable format (Word document).

Source code for data analyses and creation of publication figures are available at the following Git repositories, access to which can be requested from the authors:

<https://bitbucket.org/sakoay/acctowerscuelockphenom_2020>

<https://bitbucket.org/sakoay/publicationscripts>

<https://github.com/BrainCOGS/TankMouseAnalysis>

<https://github.com/BrainCOGS/SAKFunctions>
